# Contract Labor Reliance and Subsequent Patient Experience Performance: Evidence from U.S. Short-Term Acute Care Hospitals

**DOI:** 10.3390/healthcare14111537

**Published:** 2026-06-01

**Authors:** Bradley Beauvais, Zo Ramamonjiarivelo, Michael Mileski, Roland Shapley, Rohit Pradhan, Ramalingam Shanmugam

**Affiliations:** School of Health Administration, Texas State University, San Marcos, TX 78666, USA; zhr3@txstate.edu (Z.R.); mileski@txstate.edu (M.M.); rshapley@txstate.edu (R.S.); pradhan@txstate.edu (R.P.); shanmugam@txstate.edu (R.S.)

**Keywords:** contract labor, patient experience, HCAHPS, hospital quality, workforce composition, value-based purchasing

## Abstract

**Background/Objectives**: U.S. hospitals increasingly rely on contract labor to address persistent workforce shortages; however, the downstream implications for patient experience performance remain underexplored. This study examines whether contract labor reliance is associated with subsequent patient experience outcomes. **Methods**: Using a national sample of non-federal U.S. short-term acute care hospitals (N = 2099), this observational study evaluates whether contract labor reliance in 2024 was associated with patient experience outcomes in 2025. Contract labor reliance was measured as contract labor expense divided by total salary expense. Primary outcomes included HCAHPS Summary Star Rating and Hospital Compare Overall Rating. Multivariable ordinary least squares regression models with regional and ownership fixed effects were estimated, and quadratic specifications were used to assess nonlinear associations. Ordered logit models were also estimated as robustness checks and yielded substantively similar directional results. **Results**: Higher contract labor reliance was significantly associated with lower subsequent patient experience ratings across both outcomes (HCAHPS: β = −5.288, *p* < 0.001; Hospital Compare: β = −6.463, *p* < 0.001). Positive quadratic terms indicated a convex relationship, suggesting that marginal negative associations diminish at higher levels of reliance. Quartile-based analyses demonstrated a monotonic decline in patient experience performance across increasing levels of contract labor reliance. **Conclusions**: Contract labor reliance is significantly associated with subsequent patient experience performance. These findings should be interpreted as associations rather than causal effects and suggest that workforce composition may represent an important structural factor associated with hospital quality performance.

## 1. Introduction

Healthcare workforce instability remains one of the most pressing structural challenges confronting U.S. hospitals. Persistent shortages, heightened turnover, and burnout following the COVID-19 pandemic have driven hospitals to rely increasingly on contract and agency labor to sustain operations [1,2,3]. Hospitals operate within highly competitive labor markets in which contract labor pricing—particularly for nursing and allied health professionals—is often determined by regional supply–demand imbalances, limiting managerial discretion in wage-setting and contractual arrangements [4,5]. While this strategy provides short-term staffing flexibility, it may carry unintended quality implications that extend beyond immediate operational concerns.

Prior research examining workforce structure and hospital performance has focused primarily on aggregate labor cost intensity or agency staffing exposure in cross-sectional designs. These studies suggest that labor composition influences multiple quality domains, including safety and patient experience. However, existing work has not systematically examined whether contract labor reliance predicts subsequent patient experience performance under a lagged specification. Moreover, the nonlinear implications of workforce substitution have not been explicitly modeled. More broadly, the literature across healthcare and service management suggests a tension between cost efficiency and service quality when labor flexibility substitutes for relational continuity [6,7]. Empirical work has linked staffing instability and temporary labor use to variability in quality outcomes, though findings remain context-dependent [8,9]. This study extends prior work by isolating contract labor reliance, incorporating temporal ordering, and formally testing for curvilinear associations.

Prior research has demonstrated that aggregate hospital labor cost intensity is negatively associated with hospital quality outcomes [10]. More recently, agency staffing exposure has been linked to contemporaneous declines in multiple quality domains, including patient experience and performance-based reimbursement measures [11]. However, these studies did not isolate whether reliance on contract labor predicts subsequent patient experience performance after accounting for hospital complexity, financial condition, and market structure. Unlike our prior analyses of aggregate labor cost intensity and general agency staffing exposure, the present study isolates contract labor share as a specific structural workforce composition metric and links it to subsequent patient experience performance using a lagged specification. This study differs substantively from prior work by isolating contract labor reliance as a distinct structural construct, introducing temporal ordering, and formally testing nonlinear relationships. Although this work builds on earlier analyses, it addresses a distinct research question—whether the composition of the workforce, rather than total labor spending or generic agency exposure, is associated with future patient experience outcomes—and therefore does not simply replicate prior findings. Prior studies have largely relied on cross-sectional designs and aggregate cost measures; in contrast, this study evaluates whether staffing composition is associated with subsequent performance and whether this relationship varies across levels of contract labor intensity. This distinction directly addresses concerns regarding incremental contribution and establishes a clearer research gap.

While prior studies have examined labor cost intensity and agency staffing exposure, these approaches primarily treat labor as a financial input or contemporaneous operational condition. In contrast, this study conceptualizes contract labor share as a structural characteristic of workforce composition and evaluates its association with subsequent patient experience outcomes using temporal ordering and nonlinear modeling. This distinction shifts the analytical focus from cost magnitude to workforce structure itself.

Patient experience measures derived from the Hospital Consumer Assessment of Healthcare Providers and Systems (HCAHPS) survey have become central indicators of hospital quality and are incorporated directly into CMS value-based purchasing programs [12]. These measures reflect communication quality, responsiveness, discharge processes, and overall perceptions of care—domains highly sensitive to continuity, teamwork, and organizational integration. Given that patient experience scores directly influence hospital reimbursement through value-based purchasing mechanisms, understanding the structural determinants of these outcomes has significant financial and strategic implications.

This study builds on prior labor cost and agency staffing research [10,11] by isolating contract labor reliance as a structural organizational characteristic and evaluating its association with subsequent patient experience outcomes using temporal ordering (2024 staffing composition predicting 2025 performance). Specifically, we address three research questions: (1) Is contract labor reliance associated with subsequent patient experience performance after controlling for organizational and market characteristics? (2) Is this relationship nonlinear, suggesting threshold effects? (3) What are the practical implications for hospital workforce management and quality governance? We posit that contract labor reliance should be considered as a structural characteristic potentially associated with care quality that operates through disruptions to relational continuity, institutional knowledge, and team coordination.

## 2. Conceptual Framework

This study integrates Donabedian’s structure–process–outcome framework [13], agency theory [14,15], and high-reliability organization theory [16] to develop a theoretically grounded model linking workforce composition to patient experience outcomes.

Donabedian’s framework conceptualizes quality as emerging from structural inputs that shape care processes and ultimately outcomes [13]. Contract labor reliance represents a structural feature that may influence communication reliability, coordination, and continuity-process-level factors directly captured by patient experience metrics. Specifically, the substitution of permanent staff with temporary contract workers alters the structural composition of care delivery teams, potentially disrupting established communication patterns and standardized workflows that contribute to consistent patient experiences.

Agency theory suggests that temporary staffing arrangements may attenuate alignment between individual and organizational goals, increasing monitoring costs and reducing long-term relational investment [14,15]. Contract workers, by definition, lack the long-term employment relationship that fosters organizational commitment and alignment with institutional quality objectives. This misalignment may manifest as reduced discretionary effort in patient communication, lower engagement in quality improvement initiatives, and diminished investment in learning institution-specific protocols and patient population characteristics.

High-reliability organization theory further emphasizes stable teams, shared norms, and institutional memory as prerequisites for consistent performance in complex environments [16]. Excessive reliance on temporary staffing may undermine these attributes by fracturing the collective competence required for anticipatory problem-solving and seamless care coordination. In high-reliability organizations, team members develop shared mental models that enable them to coordinate implicitly, anticipate potential failures, and adapt rapidly to emerging challenges. Contract labor disrupts these shared mental models, potentially increasing coordination failures that patients experience as communication breakdowns, delays, or unresponsiveness.

The theoretical framework suggests two core premises. First, workforce composition represents a structural input that shapes process-level dimensions of care, particularly communication and continuity. Second, the introduction of temporary staffing into established clinical teams may produce transitional disruption effects that are not linear. Third, Public Service Motivation theory suggests that individuals in public-serving roles may be intrinsically motivated by alignment with organizational mission and patient-centered values [17]. Permanent hospital staff may exhibit stronger mission alignment and relational investment compared to contract personnel, whose temporary employment structure may attenuate such alignment.

While this study does not directly measure motivation, differences in Public Service Motivation provide a complementary mechanism linking workforce composition to patient experience outcomes. The inclusion of nonlinear specifications is theoretically informed by organizational adaptation theory, which suggests that systems undergoing structural disruption may experience initial instability followed by partial stabilization as processes adapt [18]. Taken together, Public Service Motivation and organizational adaptation theory help explain not only why contract labor reliance may be associated with patient experience, but also why that association may vary across levels of reliance rather than remain strictly linear.

Based on this integrated theoretical framework, we hypothesize that: (H1) Contract labor reliance will be negatively associated with subsequent patient experience performance. (H2) This relationship will exhibit nonlinear characteristics, with marginal effects varying across different levels of contract labor intensity. In this context, a “negative association” indicates that higher values of contract labor share correspond to lower subsequent patient experience scores.

## 3. Materials and Methods

### 3.1. Data Source and Sample

Data were obtained from Definitive Healthcare [19], a comprehensive national database that aggregates hospital-level operational, financial, and quality data from multiple sources including Medicare cost reports, hospital financial statements, and CMS quality reporting systems. The database includes non-federal short-term acute care hospitals operating in the United States. These data are derived from Medicare cost reports and audited financial statements, which follow standardized CMS reporting conventions and support cross-hospital comparability despite minor reporting variation.

Independent variables were measured in 2024 and outcomes in 2025 to establish temporal precedence and reduce concerns about reverse causation. Observations with missing data for any variable included in a given model were excluded using listwise deletion; sample sizes therefore vary by outcome. Although this approach preserves internal consistency within each model, it may introduce selection bias if hospitals with missing financial data differ systematically from the analytic sample. To assess the robustness of our findings to this assumption, we compared observable characteristics between included and excluded hospitals and re-estimated the primary specification using multiple imputation by chained equations (five imputations) with the same covariate structure. Results were substantively similar across specifications (Appendix Table A1), indicating that the primary findings are not driven by missing data patterns. While this lag structure does not eliminate reverse causality, it provides a stronger approximation of directional association than contemporaneous models.

The analytic objective is to evaluate directional association rather than to model longitudinal trends in patient experience outcomes. While multi-year panel designs would allow estimation of within-hospital changes over time, this study focuses on whether prior-year workforce composition is associated with subsequent performance differences across hospitals.

### 3.2. Variables

#### 3.2.1. Dependent Variables

The outcomes considered in the study were the 2025 HCAHPS Summary Star Rating and 2025 Hospital Compare Overall Rating. The HCAHPS Summary Star Rating ranges from 1 to 5 stars and is derived from patient responses to standardized survey questions covering communication with nurses and doctors, responsiveness of hospital staff, communication about medicines, discharge information, care transitions, cleanliness and quietness of the hospital environment, and overall hospital rating [12]. The Hospital Compare Overall Rating is a composite measure also ranging from 1 to 5 stars that integrates patient experience, clinical outcomes, safety, efficiency, and timeliness metrics. Both measures are publicly reported and are used in CMS Value-Based Purchasing programs and can influence hospital reimbursement.

#### 3.2.2. Independent Variable of Interest

The independent variable of interest was the 2024 Contract Labor Share, defined as contract labor expense divided by total salary expense. This financial-based measure captures the proportion of total compensation costs attributable to temporary or contract workers, reflecting the relative intensity of contract labor utilization. The measure includes all contract-based personnel, including clinical and non-clinical roles. While this limits role-specific interpretation, patient experience measures are most sensitive to frontline clinical interactions, suggesting that estimated associations likely reflect clinical staffing composition. A quadratic term (Contract Labor Share squared) tested for nonlinear associations to assess whether marginal effects vary across different levels of contract labor intensity. Because the contract labor measure is expense-based and includes both clinical and non-clinical roles, the mechanisms linking workforce composition to patient experience are inferred rather than directly measured.

#### 3.2.3. Control Variables

All controls were drawn from the 2024 reporting year and included case mix index, average daily census, staffed beds, Medicare days, Medicaid days, market concentration index, days cash on hand, hospital teaching status (medical school affiliation), and geographic classification (urban vs. rural). Case mix index reflects the average diagnostic complexity and resource intensity of patients treated, with higher values indicating more complex patient populations. Average daily census and staffed beds capture hospital scale and capacity utilization. Medicare days and Medicaid days represent the proportion of total patient days attributable to each payer source, controlling for payer mix and potential financial pressures. The market concentration index (Herfindahl–Hirschman Index) measures the degree of market competition, with higher values indicating more concentrated markets. Days cash on hand reflects financial liquidity and organizational financial health.

#### 3.2.4. Fixed Effects

Region and ownership fixed effects were included to control for unobserved geographic and organizational heterogeneity. Regional fixed effects account for geographic variation in labor markets, regulatory environments, and patient populations. Ownership fixed effects (government, proprietary, non-profit) control for differences in organizational mission, governance structures, and operational priorities that may influence both staffing decisions and patient experience outcomes.

#### 3.2.5. Data Preparation

Contract Labor Share was winsorized at the 1st and 99th percentiles prior to model estimation to reduce the influence of extreme outliers while preserving the majority of observed variation; the quadratic term was constructed from the winsorized Contract Labor Share. Winsorization was applied only to Contract Labor Share; other predictors were retained in their original form. We examined the distribution of all continuous covariates for extreme values. Days Cash on Hand exhibited several extreme negative and positive outliers. Sensitivity analyses were conducted using winsorized values (1st and 99th percentiles) and log-transformed specifications; results were substantively unchanged. Accordingly, the primary models retain the original scale to preserve interpretability.

### 3.3. Statistical Analysis

Multivariable OLS regression models were estimated for each outcome with robust standard errors (HC1) to account for potential heteroscedasticity and region and ownership fixed effects. Although outcome variables are ordinal, OLS models were used for interpretability and consistency with prior literature; ordered logit models were estimated as robustness checks and yielded substantively similar directional results. The general model specification was:

$$Y_{i,2025}=\beta_{0}+\beta_{1}{\mathrm{ContractShare}}_{i,2024}+\beta_{2}{\mathrm{ContractShare}}_{i,2024}^{2}+\gamma X_{i,2024}+\delta_{r}+\theta_{o}+\epsilon_{i}$$

where $Y_{i,2025}$ represents the patient experience outcome for hospital i in 2025, ${\mathrm{ContractShare}}_{i,2024}$ is the contract labor share in 2024, $X_{i,2024}$ is a vector of control variables, $\delta_{r}$ and $\theta_{o}$ represent region and ownership fixed effects respectively, and $\epsilon_{i}$ is the error term. The coefficient $\beta_{1}$ captures the linear effect of contract labor share, while $\beta_{2}$ captures the quadratic (nonlinear) effect.

To interpret the quadratic relationship, we calculated the turning point (if within the observed data range) using the formula: $\mathrm{Turning}\;\mathrm{Point}=-\beta_{1}/(2\beta_{2})$. We also computed marginal effects at representative values of contract labor share to illustrate how the relationship varies across different intensity levels.

Coefficients for region and ownership fixed effects are not reported to preserve table parsimony but were included in all models to account for geographic and governance heterogeneity. All analyses were conducted using R version 4.3.2 [20]. Statistical significance was assessed at the α = 0.05 level.

## 4. Results

### 4.1. Descriptive Statistics

Table 1 presents descriptive statistics for all variables included in the analysis.

The descriptive statistics reveal substantial variation in contract labor reliance across hospitals, with a mean of 5.8% and standard deviation of 6.0%, ranging from 0% to 46.1% (after winsorization). Patient experience performance also varied considerably, with HCAHPS Summary Star Ratings averaging 3.0 stars (SD = 0.81) and Hospital Compare Overall Ratings averaging 3.0 stars (SD = 1.09). The sample represents diverse hospital characteristics in terms of size, complexity, payer mix, and market structure, supporting generalizability to a broad cross-section of U.S. non-federal short-term acute care hospitals.

### 4.2. Regression Analysis

Table 2 reports regression results for HCAHPS Summary Star Rating.

Table 3 below reports regression results for Hospital Compare Overall Rating.

The regression results provide strong support for H1, demonstrating that contract labor reliance is negatively associated with subsequent patient experience performance across both outcome measures. The linear coefficient for contract labor share is negative and statistically significant in both models (HCAHPS: β = −5.288, *p* < 0.001; Hospital Compare: β = −6.463, *p* < 0.001), indicating that higher contract labor reliance is associated with lower patient experience ratings one year later.

Support for H2 is also evident, as the positive and statistically significant quadratic terms (HCAHPS: β = 10.051, *p* < 0.001; Hospital Compare: β = 9.792, *p* = 0.001) indicate that the relationship exhibits nonlinear characteristics.

**Figure A1:**
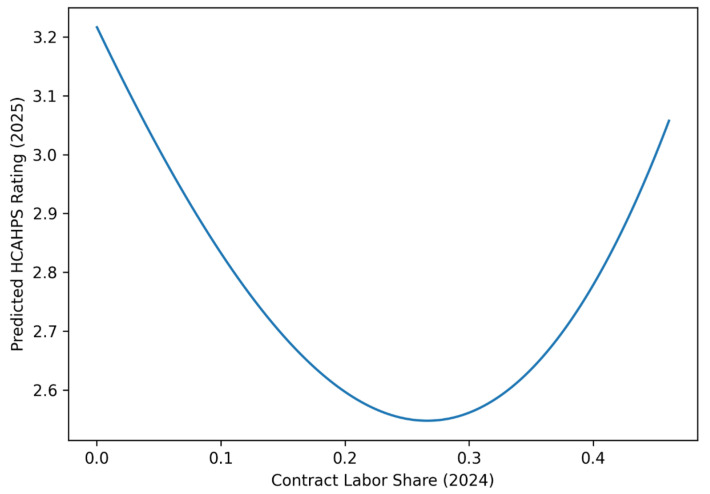
Cubic Spline Curve (HCAHPS Model).

The positive quadratic coefficient combined with a negative linear coefficient indicates a convex relationship in which the marginal negative association diminishes as contract labor share increases. The estimated turning points occur at approximately 26% contract labor share for HCAHPS and 33% for Hospital Compare, suggesting that the steepest declines in patient experience arise at low-to-moderate levels of reliance, with marginal associations attenuating at higher levels. Quartile-based specification results (Appendix Table A3) indicate a monotonic decline in HCAHPS ratings across increasing levels of contract labor reliance, with the largest negative association observed in the highest quartile. Cubic spline specifications yielded similar curvature patterns (Appendix Figure A1), further supporting the presence of nonlinear dynamics rather than a strictly linear decline. Parallel robustness checks for the Hospital Compare model produced substantively similar patterns, indicating that the observed curvature is not dependent on the specific functional form imposed by the quadratic specification.

Among control variables, several significant associations emerged. Case mix index demonstrated a positive association with patient experience in both models (HCAHPS: β = 0.529, *p* < 0.001; Hospital Compare: β = 0.563, *p* < 0.001), suggesting that hospitals treating more complex patients may invest in enhanced coordination systems that benefit patient experience. Medicare days exhibited positive associations (HCAHPS: β = 0.407, *p* = 0.022; Hospital Compare: β = 1.411, *p* < 0.001), potentially reflecting alignment between Medicare quality incentives and organizational attention to patient-facing performance. Market concentration showed a positive association with HCAHPS ratings (β = 0.309, *p* < 0.001) but was not statistically significant for Hospital Compare Overall Rating (β = −0.035, *p* = 0.697). Days cash on hand was not statistically significant in the HCAHPS model (*p* = 0.083) but demonstrated a statistically significant positive association in the Hospital Compare model. Teaching hospitals exhibited significantly lower patient experience ratings in both models (HCAHPS: β = −0.209, *p* < 0.001; Hospital Compare: β = −0.238, *p* < 0.001), while urban hospitals were associated with lower HCAHPS ratings (β = −0.220, *p* < 0.001) and a weaker, non-significant association for Hospital Compare (β = 0.132, *p* = 0.072). The explained variance (R^2^ = 0.285 for HCAHPS; R^2^ = 0.183 for Hospital Compare) indicates moderate explanatory power, though substantial unexplained variance remains, suggesting additional unmeasured organizational and cultural factors also influence these outcomes.

## 5. Discussion

This study provides evidence that contract labor reliance is associated with subsequent patient experience performance across two national quality indicators. By introducing temporal ordering and isolating workforce composition from aggregate labor intensity, the analysis strengthens inference relative to prior cross-sectional research [10,11]. The findings indicate that contract labor is associated with measurable downstream performance differences after accounting for case mix, hospital scale, payer mix, market concentration, liquidity, and both regional and ownership fixed effects. Findings should be interpreted as associations rather than causal effects. The use of a lagged design, a contract-labor-specific exposure, and formal tests of nonlinearity were not features of the earlier studies and therefore represent a substantive extension rather than a repackaging of previous results. The findings should not be interpreted as indicating that contract labor is inherently detrimental. Rather, outcomes likely depend on how contract labor is integrated and managed within organizational systems. Accordingly, the present study should be viewed as an extension of prior workforce composition research that further evaluates temporal ordering and nonlinear association patterns rather than as a wholly separate line of inquiry.

The nonlinear specification provides particularly important insight, indicating a convex relationship. While recent work by Beauvais et al. [11] established a linear, contemporaneous negative association between agency staffing and quality, our findings suggest that the association between contract labor reliance and patient experience is more complex over time. The positive quadratic term implies that the marginal negative association is strongest at lower-to-moderate levels of reliance and diminishes as reliance increases. The estimated turning point occurs at approximately 26% contract labor share for HCAHPS ratings and 33% for Hospital Compare Overall Rating. This pattern is consistent with—but does not definitively establish—a transitional disruption mechanism. Potential explanations include coordination strain, communication instability, and disruptions in relational continuity; however, these mechanisms were not directly measured in the present study. At lower and moderate levels of reliance, the introduction of contract labor into established clinical teams may generate coordination strain, communication instability, and relational discontinuity. At higher levels of reliance, hospitals may adapt structurally to a more transactional staffing model, leading to attenuation of further declines in patient experience. Alternatively, hospitals with very high contract labor reliance may represent a distinct organizational subtype that has implemented systematic processes, training protocols, and supervisory structures tailored to predominantly temporary workforces. While these interpretations remain inferential, the results indicate that the relationship is not strictly linear and that workforce substitution effects vary across the distribution of contract labor intensity.

This erosion of patient experience at low-to-moderate levels of reliance may be explained by the weakening of relational cohesion within clinical units. Research on nursing practice environments demonstrates that high-quality care settings rely on stable professional relationships, shared governance, and institutional support structures that reinforce coordination and trust [21,22]. Permanent staff accumulate institutional memory and relational capital that facilitate implicit communication and anticipation of patient needs. When contract labor is introduced into established teams, these shared understandings may be disrupted, potentially producing coordination strain that patients perceive as unresponsiveness or lack of empathy. The initial introduction of contract labor may therefore be particularly destabilizing, as team routines are partially disrupted without yet being structurally reconfigured. At moderate levels of reliance, hospitals may experience the greatest disruption-established teams are fractured, but organizational processes have not yet adapted to a predominantly temporary workforce model.

The magnitude of these associations is substantively meaningful. Given that HCAHPS Summary Star Ratings range from one to five stars and directly influence publicly reported quality profiles and value-based purchasing adjustments, even moderate shifts in predicted ratings may have financial and reputational implications. To illustrate the practical significance, consider a hospital moving from the 25th percentile of contract labor share (approximately 2%) to the 75th percentile (approximately 10%). Based on the updated HCAHPS model coefficients (β_1_ = −5.288; β_2_ = 10.051), the linear component predicts a decline of −5.288 × 0.08 = −0.423 stars. The quadratic adjustment partially offsets this decline: 10.051 × (0.10^2^ − 0.02^2^) ≈ 0.096. The net predicted change is therefore approximately −0.327 stars. Relative to the sample mean of approximately 3.0 stars, this represents an 11% decline. For hospitals clustered near star-category thresholds, even a one-third star shift may meaningfully affect publicly reported ratings and reimbursement adjustments. Because patient experience accounts for 25% of the CMS Value-Based Purchasing (VBP) domain score, structural reliance on contract labor may carry indirect reimbursement implications in addition to direct staffing costs.

Secondary findings reinforce the structural interpretation. Alternative explanations include regional labor shortages, pandemic recovery dynamics, and financial distress, which may jointly influence both contract labor reliance and patient experience outcomes. Liquidity (days cash on hand) exhibits a positive association with Hospital Compare outcomes, suggesting that financially stable hospitals may be better positioned to integrate and supervise temporary staff through stronger onboarding infrastructure, managerial oversight, and service-recovery systems. Market concentration shows a positive association with HCAHPS ratings but is not statistically significant for Hospital Compare Overall Rating. This divergence suggests that structural advantages in more concentrated markets may affect patient experience domains specifically, rather than general quality performance.

Case mix index demonstrates a positive association in both models, consistent with the possibility that higher-complexity hospitals employ more formalized coordination systems that support communication and responsiveness.

Days cash on hand was not statistically significant in the HCAHPS model (*p* = 0.083), but demonstrated a statistically significant positive association in the Hospital Compare model (*p* = 0.002), consistent with the coefficients reported in Table 2 and Table 3.

Teaching hospitals are associated with lower patient experience ratings in both models, while urban hospitals exhibit lower HCAHPS ratings and a weaker association with Hospital Compare. These associations likely reflect structural complexity and academic mission demands rather than workforce composition alone. These findings also suggest that structural and organizational complexity may influence patient-facing performance independently of workforce composition. Importantly, the nonlinear contract labor relationship remains statistically significant even after controlling for these structural characteristics, indicating that the observed association is not solely attributable to academic status or urban labor market constraints.

A particularly important finding is the persistence of the contract labor association across both HCAHPS-specific ratings and the broader Hospital Compare Overall Rating. This consistency suggests that workforce composition affects fundamental care delivery processes rather than being confined to specific survey domains. The fact that contract labor reliance is associated with both patient experience and overall quality ratings implies broader organizational implications beyond patient satisfaction alone.

### Theoretical Implications

This study contributes to theory by extending Donabedian’s structure–process–outcome framework by operationalizing workforce composition as a measurable structural input associated with patient-centered outcomes [13]. Second, it contributes to agency theory by highlighting how temporary employment relationships may weaken alignment between individual and organizational objectives [14,15]. Third, the findings are consistent with organizational adaptation theory, suggesting nonlinear responses to structural disruption [18]. Finally, Public Service Motivation theory provides a complementary explanation through differences in intrinsic motivation across staffing types [17]. Taken together, the results provide preliminary evidence of an association between workforce composition and patient experience outcomes and should be interpreted as indicative rather than definitive.

## 6. Practical Implications

Hospital leaders may consider tracking contract labor share not only as a purely financial metric but also as a potential structural quality indicator and incorporating it into routine performance dashboards alongside clinical and patient experience outcomes. Rather than relying on ad hoc staffing adjustments, organizations may benefit from establishing internal, context-specific monitoring thresholds that prompt review when contract labor reliance increases beyond typical baseline levels. Because the marginal negative effects appear strongest during transitional phases of increasing reliance—particularly below the estimated turning point of approximately 26–33%—hospitals should be attentive to moderate increases in contract staffing that may destabilize established care routines before organizational adaptation occurs.

Establishing internal guardrails for sustained reliance, strengthening onboarding and supervision processes for temporary staff, and targeting handoff and communication reliability may help mitigate declines in patient experience, although these operational strategies were not directly tested in this study. Practical interventions could include: (1) standardized onboarding protocols that rapidly orient contract workers to institution-specific workflows and communication norms; (2) structured preceptor or mentorship pairings between contract staff and experienced permanent clinicians; (3) enhanced handoff and structured communication tools designed to compensate for reduced institutional memory; and (4) systematic monitoring of patient experience metrics stratified by unit-level contract labor intensity to enable targeted interventions.

## 7. Study Limitations

This study has several recognized limitations. To begin with, this research is observational and cannot establish causality. Although temporal ordering reduces concerns about simultaneity, unobserved time-varying factors may influence both contract labor reliance and patient experience outcomes. Examples include leadership transitions, concurrent quality improvement initiatives, service-line restructuring, and local labor market shocks. While we incorporated region, ownership, teaching status, and urban/rural designation to account for structural heterogeneity, residual confounding may persist. Reverse causality remains possible, as hospitals with lower patient experience performance may increase reliance on contract labor. Future research employing quasi-experimental designs—such as difference-in-differences approaches leveraging exogenous staffing shocks or instrumental variable strategies based on regional labor supply disruptions—could strengthen causal inference.

Because the analytic design links 2024 exposures to 2025 outcomes using a single lag rather than a multi-year panel, the study does not model within-hospital temporal dynamics. Future research using longer panel designs with hospital fixed effects could address this limitation more directly.

The contract labor measure is expense-based and does not differentiate between clinical and non-clinical roles. Inclusion of non-clinical contract labor (e.g., information technology, food service, environmental services, administrative support) may attenuate the estimated association between contract labor reliance and patient experience. Because HCAHPS domains are particularly sensitive to nursing and direct clinical communication, a role-specific measure of contract nursing intensity would likely produce more precise and potentially larger effect estimates. Future research should disaggregate contract labor by occupational category to better isolate clinically relevant mechanisms. In addition, because contract labor share is expense-based, it conflates staffing levels with wage differentials and pricing variation, which may bias estimates if high contract rates reflect labor market conditions rather than changes in headcount.

Important confounders—including nurse staffing ratios, staff turnover, organizational culture, leadership quality, and electronic health record adoption—were not available in the current dataset and may influence both staffing decisions and patient experience outcomes.

Listwise deletion was used in the primary models to maintain specification consistency. Although multiple imputation analyses (Appendix Table A1 and Table A2) produced substantively similar estimates, hospitals with incomplete data may differ in unobserved ways from those included in the analytic sample. Additionally, the absence of hospital fixed effects limits control for time-invariant heterogeneity.

Finally, Hospital Compare Overall Rating includes patient experience components, introducing partial overlap with HCAHPS outcomes. Although quadratic modeling captures curvature in the contract labor–quality relationship, it does not identify a precise inflection point with high statistical certainty. The estimated turning points should therefore be interpreted cautiously, particularly given the relatively small number of hospitals with very high contract labor shares.

## 8. Conclusions

Contract labor reliance in 2024 is significantly associated with patient experience performance in 2025. The nonlinear association suggests that declines in patient-perceived quality are most acute during the initial introduction of contract labor into a predominantly permanent workforce, consistent with the disruption of established team norms and the erosion of social capital before adaptive organizational structures emerge. These findings extend prior research on labor cost intensity and agency staffing by isolating workforce composition as an independent structural factor associated with patient-centered quality outcomes. From a strategic perspective, workforce composition may be considered for inclusion in hospital quality governance frameworks. Hospital administrators should carefully weigh the short-term operational flexibility of agency labor against the potential long-term erosion of the relational capital required to deliver high-quality patient experiences. Moreover, the financial implications extend beyond direct contract labor premiums to include opportunity costs associated with reduced value-based purchasing revenues, suggesting that the true cost of contract labor reliance may be substantially higher than budget projections indicate. Future research should examine specific mitigation strategies, optimal contract labor thresholds by hospital type and setting, and the long-term trajectory of hospitals transitioning from low to high contract labor models.

## Figures and Tables

**Table 1:** Descriptive statistics for the HCAHPS analytic sample (N = 2099).

Variable	N	Mean	SD	Min	Max
Dependent Variables					
HCAHPS Summary Star Rating (2025)	2099	3.007	0.805	1.000	5.000
Hospital Compare Overall Rating (2025)	2012	3.039	1.086	1.000	5.000
Independent Variable of Interest					
Contract Labor Share (2024)	2099	0.058	0.060	0.000	0.461
Continuous Control Variables					
Case Mix Index (2024)	2099	1.765	0.350	0.998	4.986
Average Daily Census (2024)	2099	163.221	193.499	1.117	2437.046
Staffed Beds (2024)	2099	232.170	205.813	9.000	2247.000
Medicare Days (2024)	2099	0.255	0.096	0.002	0.649
Medicaid Days (2024)	2099	0.077	0.077	0.000	0.633
Market Concentration Index (2024)	2099	0.356	0.328	0.020	1.000
Days Cash on Hand (2024)	2099	43.866	165.232	−1185.931	3406.276
Teaching Hospital	2099	0.508	0.5	0.0	1.0
Urban Hospital	2099	0.775	0.418	0.0	1.0
Organizational Structure Control Variables-Region					
Southeast Region	551	0.263	-	-	-
Midwest Region	531	0.253	-	-	-
Northeast Region	395	0.188	-	-	-
West Region	353	0.168	-	-	-
Southwest Region	269	0.128	-	-	-
Organizational Structure Control Variables-Ownership					
Government Ownership	263	0.125	*-*	*-*	*-*
Proprietary Ownership	371	0.176	-	-	-
Non-Profit Ownership	1465	0.698	-	-	-

**Table 2:** Regression results for HCAHPS Summary Star Rating. N = 2099; R^2^ = 0.285. Model includes region and ownership fixed effects (not reported). Dependent variable: HCAHPS Summary Star Rating (2025). All independent variables measured in 2024. Robust standard errors (HC1) reported.

Variable	Coefficient	SE	*p*-Value
Contract Labor Share (2024)	−5.288	0.571	<0.001
Contract Labor Share ^2^ (2024)	10.051	1.980	<0.001
Case Mix Index (2024)	0.529	0.055	<0.001
Average Daily Census (2024)	0.000	0.000	0.024
Staffed Beds (2024)	−0.001	0.000	<0.001
Medicare Days (2024)	0.407	0.177	0.022
Medicaid Days (2024)	−0.204	0.208	0.327
Market Concentration Index (2024)	0.309	0.060	<0.001
Days Cash on Hand (2024)	0.000	0.000	0.083
Teaching Hospital	−0.209	0.035	<0.001
Urban Hospital	−0.220	0.049	<0.001

**Table 3:** Regression results for Hospital Compare Overall Rating. N = 2012; R^2^ = 0.183. Model includes region and ownership fixed effects (not reported). Dependent variable: Hospital Compare Overall Rating (2025). All independent variables measured in 2024. Robust standard errors (HC1) reported.

Variable	Coefficient	SE	*p*-Value
Contract Labor Share (2024)	−6.463	0.855	<0.001
Contract Labor Share ^2^ (2024)	9.792	3.005	0.001
Case Mix Index (2024)	0.563	0.094	<0.001
Average Daily Census (2024)	0.001	0.000	<0.001
Staffed Beds (2024)	−0.001	0.000	<0.001
Medicare Days (2024)	1.411	0.260	<0.001
Medicaid Days (2024)	−0.332	0.305	0.278
Market Concentration Index (2024)	−0.035	0.089	0.697
Days Cash on Hand (2024)	0.000	0.000	0.002
Teaching Hospital	−0.238	0.052	<0.001
Urban Hospital	0.132	0.073	0.072

## Data Availability

Licensing Restrictions apply to the availability of these data. Data were obtained from Definitive Healthcare and are available from the authors with the permission of Definitive Healthcare.

## Appendix

### Appendix A.1. Comparison of Listwise Deletion and Multiple Imputation Estimates (HCAHPS Model)

Appendix Table A1 presents a side-by-side comparison of regression coefficients and standard errors obtained using listwise deletion and multiple imputation by chained equations (five imputations). Pooled estimates for multiple imputation were calculated using Rubin’s rules. The similarity of coefficients and standard errors across estimation approaches suggests that listwise deletion did not materially influence the estimated associations.

**Table A1:** Comparison of Listwise Deletion and Multiple Imputation Estimates (HCAHPS Model).

Variable	Listwise Coef.	Listwise SE	MI Coef. (Pooled)	MI SE (Pooled)
Intercept	2.143	0.075	2.143	0.075
Contract Labor Share (2024)	−5.806	0.462	−5.242	0.444
Contract Labor Share ^2^ (2024)	11.546	1.238	11.935	1.185
Case Mix Index (2024)	0.557	0.036	0.546	0.034
Average Daily Census (2024)	0.001	0.000	0.001	0.000
Staffed Beds (2024)	−0.002	0.000	−0.002	0.000
Medicare Days (2024)	1.021	0.137	1.006	0.131
Medicaid Days (2024)	−0.338	0.161	−0.322	0.154
Market Concentration Index (2024)	0.475	0.051	0.567	0.045
Days Cash on Hand (2024)	0.000	0.000	0.000	0.000
Teaching Hospital	−0.209	0.054	−0.214	0.052
Urban Hospital	−0.220	0.048	−0.227	0.046

Note: Models include teaching status and urban designation; region and ownership fixed effects are excluded in appendix models for comparability across imputation procedures. Pooled estimates computed using Rubin’s rules (five imputations).

### Appendix A.2. Comparison of Listwise Deletion and Multiple Imputation Estimates (Hospital Compare Model)

Appendix Table A2 presents a side-by-side comparison of regression coefficients and standard errors for the Hospital Compare Overall Rating model estimated using listwise deletion and multiple imputation (five imputations). As with the HCAHPS model, coefficient magnitudes and standard errors are substantively similar across approaches, indicating that missing data handling does not materially alter the conclusions.

**Table A2:** Comparison of Listwise Deletion and Multiple Imputation Estimates (Hospital Compare Model).

Variable	Listwise Coef.	Listwise SE	MI Coef. (Pooled)	MI SE (Pooled)
Intercept	2.016	0.178	2.048	0.099
Contract Labor Share (2024)	−5.485	0.868	−6.711	0.587
Contract Labor Share ^2^ (2024)	6.506	3.067	14.825	1.565
Case Mix Index (2024)	0.550	0.091	0.617	0.045
Average Daily Census (2024)	0.001	0.000	0.001	0.000
Staffed Beds (2024)	−0.001	0.000	−0.001	0.000
Medicare Days (2024)	1.830	0.258	1.595	0.173
Medicaid Days (2024)	−0.437	0.306	−0.702	0.203
Market Concentration Index (2024)	−0.102	0.075	−0.156	0.059
Days Cash on Hand (2024)	0.001	0.000	0.001	0.000
Teaching Hospital	−0.238	0.061	−0.245	0.058
Urban Hospital	0.132	0.073	0.125	0.070

Note: Models include teaching status and urban designation; region and ownership fixed effects are excluded in appendix models for comparability across imputation procedures. Pooled estimates computed using Rubin’s rules (five imputations).

### Appendix A.3. Quartile Specification Results (HCAHPS Model)

**Table A3:** Quartile Specification Results (HCAHPS Model).

Quartile (Ref = Q1)	Coefficient	Std. Error	*p*-Value
Quartile 2 (vs. Q1)	−0.092	0.046	0.045
Quartile 3 (vs. Q1)	−0.217	0.046	<0.001
Quartile 4 (vs. Q1)	−0.426	0.046	<0.001
